# The use of group dynamics strategies to enhance cohesion in a lifestyle intervention program for obese children

**DOI:** 10.1186/1471-2458-9-277

**Published:** 2009-07-31

**Authors:** Luc J Martin, Shauna M Burke, Sheree Shapiro, Albert V Carron, Jennifer D Irwin, Robert Petrella, Harry Prapavessis, Kevin Shoemaker

**Affiliations:** 1 The University of Western Ontario, London, Ontario, Canada

## Abstract

**Background:**

Most research pertaining to childhood obesity has assessed the effectiveness of preventative interventions, while relatively little has been done to advance knowledge in the treatment of obesity. Thus, a 4-week family- and group-based intervention utilizing group dynamics strategies designed to increase cohesion was implemented to influence the lifestyles and physical activity levels of obese children.

**Methods/Design:**

This paper provides an overview of the rationale for and implementation of the intervention for obese children and their families. Objectives of the intervention included the modification of health behaviors and cohesion levels through the use of group dynamics strategies. To date, a total of 15 children (7 boys and 8 girls, mean age = 10.5) and their families have completed the intervention (during the month of August 2008). Physiological and psychological outcomes were assessed throughout the 4-week intervention and at 3-, 6-, and 12-month follow-up periods.

**Discussion:**

It is believed that the information provided will help researchers and health professionals develop similar obesity treatment interventions through the use of evidence-based group dynamics strategies. There is also a need for continued research in this area, and it is our hope that the Children's Health and Activity Modification Program (C.H.A.M.P.) will provide a strong base from which others may build.

## Background

Canadian children are becoming progressively overweight and obese. In 2004, 26% of children and adolescents aged 2 to 17 were either obese or overweight [1]. The prevalence of overweight youth ages 17 and under has doubled in the last 25 years, while obesity alone has tripled [1]. These rising trends are alarming for at least two reasons. One is that they are associated with numerous detrimental physical and psychosocial outcomes including, but not limited to, increased risk of cardiovascular disease [2], hyperlipidemia, hypertension, and social discrimination [3]. A second is the likelihood that childhood obesity will continue into adulthood and increase the risk of the onset of type II diabetes [4,5]. In order to combat these potential complications, it is extremely important to intervene at an early age rather than allow the problem to continue into adulthood. During childhood, lifestyle patterns are not as well established as compared to adults. Children may be more open to behavior change and researchers and health care practitioners have a greater opportunity to use family members as social support systems.

A question that does arise is how best to address the problem. A significant amount of research has focused on the *prevention *of obesity in youth [6-9]; relatively less to its *treatment*. It is clear that effective interventions must be developed to prevent obesity in youth, but considering that in 2004 roughly 1 out of 4 Canadian children were considered to be obese or overweight [1], treatment is a growing concern.

In 2003, a systematic (Cochrane) review was conducted by Summerbell and colleagues to assess the effects of lifestyle (i.e., diet, physical activity and/or behavioral therapy) interventions lasting a minimum of 6 months designed to treat obesity in children [3]. Of the 18 randomized controlled trials included in the review, five (*n *= 245 participants) focused on the impact of changes in physical activity and sedentary behavior, two (*n *= 107 participants) compared problem-solving with usual care behavioral therapy, nine (*n *= 399 participants) compared other behavioral therapies, and two (*n *= 224 participants) compared cognitive behavioral therapy with relaxation. Most studies contained a small number of participants (i.e., at least one group in most studies contained less than 23 children), "drawn from homogenous, motivated groups in hospital settings and so generalisable evidence from them is limited" (p. 1). Thus, Summerbell and colleagues concluded that limited quality data exist with regard to obesity intervention programs for children. They also noted a scarcity of studies focusing on obesity in children that: (a) include long-term outcomes beyond one year; (b) assess psychosocial aspects of health; and (c) utilize cost-effective, community-based programs [3].

More recently, a meta-analysis of 14 randomized controlled trials was undertaken by Wilfley and colleagues to evaluate the efficacy of lifestyle interventions (defined as any combination of diet, physical activity, and/or behavioral treatment recommendations) that targeted overweight youth [10]. The number of participants in the studies ranged from 15 to 94 (in total, including treatment and control groups) with an overall mean age of 11.5 years. Treatment length ranged between 1.4 weeks and 7 months, with participants in the lifestyle interventions receiving an average of 18.3 sessions. In terms of weight status, the authors found significant large positive treatment effects (effect sizes from 0.48 – 0.75) for participants in the intervention groups when compared with wait-list control or information-only control groups, both following treatment and at follow-up. Interestingly, treatment length was not found to be a significant moderator of treatment effects, resulting in the conclusion by Wilfley and colleagues that "the optimal level of treatment contact and duration for pediatric populations has yet to be established" (p. 529). Additional recommendations were similar to those advanced by Summerbell and colleagues [3], and included: (a) incorporating follow-up assessment time points at 1-year and ideally, 2 years post-intervention; and (b) examining the effectiveness of lifestyle interventions in relation to other indices of health and psychosocial functioning for overweight and obese children [10].

It is interesting to note that only 5 (28%) of the 18 studies included by Summerbell and colleagues (2003) used physical activity as an independent variable in their interventions, compared to 12 (86%) of the 14 studies included by Wilfley and colleagues (2007). As was pointed out on the Obesity Canada website :

There are many contributing factors to obesity: activity levels, diet, genetic, metabolic, environmental, social, economic, psychological, behavioral and biological .... However, *inactivity *and *poor diet *are the two most important contributing factors to excessive weight gain.

Additionally, according to the American Dietetic Association (ADA) [11], several factors should be addressed in order to successfully decrease the prevalence of pediatric overweight and obesity. Such factors include family-based intervention programs that include the promotion of physical activity, parent training/modeling, behavioral counseling, and nutritional education. Parents and siblings represent the primary social learning environment with regards to eating and physical activity for children [12]. In fact, parental involvement is seen as essential to the success of obesity interventions by Epstein and Wing [13] because: (a) obesity runs in families, and attempting to intervene with only one family member while the others are exhibiting contradictory behaviors is unrealistic; (b) parental behaviors that facilitate overeating and inactivity are detrimental; and (c) parental support may be necessary in changing children's behaviors. The ADA also pointed out the absence of and need for evidence-based community interventions [11]. As such, in order to support healthy lifestyles, the ADA has recommended innovative lifestyle interventions involving the previously mentioned family-based programs as the most feasible way to reach the greatest number of children and their families in community settings.

Past research has shown physical activity to have a positive effect on many of the physical health problems that are prevalent in obese populations including cardiovascular health problems [14], some cancers [15], depression [16,17], and anxiety [18]. However, a question that remains is how to effectively increase–and sustain–physical activity levels in obese youth.

There is compelling evidence that interventions based on sound group dynamics principles are particularly effective for both compliance and adherence to physical activity interventions across the age span and with different populations including older adults [19-22], post-natal women [23], and female university students [24]. This is not surprising from a theoretical perspective. For example, Baumeister and Leary [25] provided a compelling argument that humans have a fundamental need to belong, and to form interpersonal relationships with others. Satisfying this need requires:

frequent, affectively pleasant interactions with a few other people ... and these interactions must take place in the context of a temporally stable and enduring framework of affective concern for each other's welfare. (p. 497)

Also from a theoretical perspective, it has been suggested that the genetic composition of humans has changed relatively little since the appearance of *homo sapiens *40,000 years ago [26]. Thus, evolutionary psychologists [27], drawing on the work of Charles Darwin (who suggested that those individuals who had the greatest propensity for living in groups had the greatest likelihood of survival and reproduction over the short and long term) consider individuals to have a natural affinity for group membership.

In an overview of the question of effective contexts for interventions designed to increase physical activity, Carron and Burke (2005) offered four sources of evidence that provided support for interventions that use the power of group influence [28]. The first included the theoretical propositions discussed above. Second, the group has been used successfully by health professionals attempting to aid in the reduction of numerous maladaptive lifestyle behaviors such as addictions, compulsions, and dependencies (e.g., gambling, alcohol, drug, and sexual addictions). Third, adherence and compliance behaviors are superior when group dynamics principles are used to develop a true group environment (i.e., in which any number of group dynamics strategies are implemented to enhance the cohesiveness of the group). Fourth, across the life span from university participants to older adults (> 65 years), participants show a preference for group contexts (i.e., individuals of all age groups would rather exercise with others in their own age cohort).

Given the strong theoretical and empirical support for interventions based on group dynamics principles, our research team conducted a pilot project to gain insight into the effectiveness of a 4-week group-based lifestyle intervention (referred to hereafter as C.H.A.M.P., an acronym for the Children's Health and Activity Modification Program) for obese children and their families. C.H.A.M.P. was developed based on group dynamics principles, the available literature, and the recommendations for effective interventions outlined above. In August, 2008, 15 obese children (aged 8–14) and their families were introduced to a number of group-based intervention components including: (1) physical activity; (2) behavior modification counselling; (3) dietary counselling; (4) weekly education sessions for families that targeted behavior modification strategies, physical activity, and nutrition in the home environment; and (5) post-intervention support for children and family members. The specific objectives of C.H.A.M.P. are to improve: (a) physical activity levels (measured using accelerometers and self-report questionnaires); (b) psychosocial outcomes (e.g., health-related quality of life, self-efficacy, perceptions of belongingness); (c) physiological outcomes (e.g., *z*-BMI, body fat percentage, lean body mass); and (d) dietary behaviors.

The general purpose of the present paper is to outline the nature of C.H.A.M.P. as it was delivered in the first year of the 2-year pilot project, with a specific focus on the group dynamics strategies that were utilized with: (a) obese children, and (b) their parents or guardians. As a secondary purpose, an instrument designed to assess obese children's perceptions of the degree of cohesiveness (unity) within the program as a whole, within subgroups of participants, and within counselor directed units is discussed.

## Methods

### What is C.H.A.M.P.?

As indicated above, C.H.A.M.P. is a community- and family-based lifestyle intervention program for obese children and their families. To our knowledge, C.H.A.M.P. is the only research-based intervention in Canada for this target population that has as its foundation the establishment (based on group dynamics strategies) of *true *groups. Our intent is to use the social influence of these true groups to effect changes in physical activity and diet, as well as numerous additional physiological and psychosocial outcomes. Other important aspects of our intervention include weekly family-based educational sessions and post-intervention group support.

As mentioned above, the primary objective of the study is to increase obese children's physical activity levels, as research has shown that sustained physical activity involvement is critical for long-term weight control [29]. Since research has documented the difficulties of physical activity adherence among children [30], the proposed study aimed at increasing physical activity behaviors in the camp *and *home environments both during and following the intervention.

#### Children's component

Children attended camp for the month of August, 2008 on weekdays (for a total of 19 days) from 9 am until 4 pm. During the 4-week program, the children engaged in a variety of fun-based physical activities. Each weekday consisted of a combination of at least six education and physical activity sessions, plus 30 minutes for lunch. Both variety and consistency were incorporated into the schedule–on most days children completed one of each of the following sessions: education (e.g., related to physical activity, nutrition, anti-bullying, etc.), aerobics (e.g., dance, yoga, etc.), water-based exercise (e.g., water polo, synchronized swimming, etc.), circuit-based or resistance training exercise (e.g., Thera-Band^® ^exercise training, machine-based circuits, etc.), and games or sports (e.g., soccer, floor hockey, dodgeball, etc.). Physical activities, education sessions, and team building activities centered on the 'theme' of the week; themes included Sports Week, Healthy Eating Week, Olympics Week, and Adventure Week. The schedule varied on Fridays in that the program was comprised of an activity-based field trip and a movie. Also, a C.H.A.M.P. Talent Show took place on the last day of camp. The child-based portion of the intervention took place at the Canadian Centre for Activity and Aging in London, Ontario. In addition, the children spent several mornings per week at the local YMCA to use the exercise equipment, swimming pool, gymnasium, and the "Y-ired Zone", a physical activity-based environment for children and youth that combines interactive computer technology with exercise.

#### Family component

Families attended weekly group-based educational sessions on four consecutive Saturdays from 10 am until 2 pm. Sessions focused on nutrition education (including an in-store supermarket tour and discussions related to portion sizes, label reading, and menu planning), healthy parenting (in which effective parenting skills and issues such as self-esteem and coping with food-related issues were discussed), diabetes education (using virtual anatomy technology which enabled family members to "tour" the human body and gain an in-depth "view" of the complications associated with diabetes), anti-bullying (led by a parent support and advocacy organization that targets bullying in schools), family goal setting (in which family members worked together to set group goals and signed a family contract), and life coaching (in which the creation of a positive and self-esteem-enhancing family environment was discussed, in addition to the value of shifting perspectives on challenging situations). All speakers discussed issues or problems related to obesity in youth and attempted to provide helpful solutions for the families involved.

Thirty minutes per session were allotted for "family picnic time", and a pot-luck lunch took place following the last session. Although the children did not attend all family-based education sessions, parents and guardians were encouraged to bring their children (including siblings) to engage in supervised and structured physical activities. All sessions took place at The University of Western Ontario, and parking was free for C.H.A.M.P. participants.

#### Program staff and volunteers

A relatively large number of staff and volunteers were involved in the organization and implementation of the intervention. The Principal Investigator, Project Coordinator, and Research Assistant ensured the smooth operation of the child and family-based portions of the intervention, and were responsible for the collection of data and supervision of the programs. A total of six program counselors, each of whom was either a university student or classroom teacher, ran the child-based portion of the intervention. Each day, two volunteers attended the camp to assist the counselors. Guest speakers for the education and physical activity sessions for the children included: the Principal Investigator, a Special Constable from the Campus Community Police, varsity and professional athletes and coaches, a Public Health Dietitian, and a Certified Co-Active Life Coach. During the weekend sessions for families, counselors and volunteers organized and supervised physical activities with the children, while guest speakers and C.H.A.M.P. research staff conducted the family training sessions. Speakers for the family sessions included the Principal Investigator, a Public Health Dietitian, an Exercise Physiologist, a Psychotherapist, an anti-bullying expert, and a Certified Co-Active Life Coach.

#### Program cost

The fee for the 4-week intervention (plus all post-program support sessions) was $200.00 (CDN). In addition to including all program activities, the fee contributed toward the cost of bussing from Monday to Friday, C.H.A.M.P. paraphernalia (e.g., backpack, pen, t-shirt, prizes, etc.), a one-month family membership at the YMCA, and all field trip fees. Lunches or snacks were not included in the cost of the program because part of the educational sessions focused on planning and preparing healthy meal choices.

#### Follow-up group support

A strategy used to ensure the success of the intervention was the use of "C.H.A.M.P. Booster Sessions". These group support sessions were offered at various locations (The University of Western Ontario, YMCA, other community-based facilities) once every two months for one year following the formal 4-week intervention. These follow-up sessions included both children and family members and were created to: (1) maintain social contact among children and family members following completion of the formal intervention; and (2) re-iterate, emphasize, and provide new information and resources pertaining to behavior modification strategies, physical activity and healthy food choices. Sessions included activity-based (i.e., curling, dance, aerobic activity) classes for children and family members, a healthy cooking demonstration with a Public Health Dietitian, a follow-up session with a Psychotherapist, a family goal setting workshop, a pot-luck lunch and healthy recipe exchange, and group discussions related to strategies for overcoming barriers to making healthy food choices and engaging in regular physical activity. The booster sessions also served as a means of sharing research-based results in order to bring families up-to-date on the children's progress throughout the intervention and at the follow-up assessment periods. Additional means of follow-up support for families included bi-monthly C.H.A.M.P. newsletters and regular follow-up telephone calls and reminders.

### Participants

Ethical approval for the study was obtained from the Health Sciences Research Ethics Board at the University of Western Ontario. Children were eligible to participate in the program if they: (a) were between the ages of 8 and 14; (b) had a body mass index (BMI) greater than or equal to the 95^th ^percentile for their age and sex; and (c) provided the researchers with signed written consent forms from both themselves and their guardians. Furthermore, in accordance with our institution's Health Sciences Research Ethics Board guidelines for Research Exercise Protocols, all children were assessed by a Pediatrician and cleared for exercise participation. A total of 16 children (50% female) with a mean age of 10.5 years and their families agreed to participate in the program. One child left the program during the second week of camp due to behavioral and family issues, and one child dropped out of the program at the 6-month follow-up after completing the formal intervention.

### Recruitment

Recruitment of participants for the 2008 C.H.A.M.P. program was accomplished through several outlets. Advertisements were placed in local newspapers and broadcasted on a local radio station. Posters were displayed in local libraries, community centers, hospitals and family medical clinics, and a one-page information sheet was faxed to all family physicians with a fax machine in London, Ontario. For the families who participated in C.H.A.M.P., the most successful means of recruitment proved to be local newspaper advertisements (*n *= 10 inquires), followed by radio advertisements (*n *= 3 inquires), posters (*n *= 3 inquires), and word-of-mouth (*n *= 2 inquires).

The present report focuses on the first year of C.H.A.M.P. which was intended to be a pilot project. As such, only a minimal number of participants (*N *= 16) were recruited–a power limitation. A power calculation was undertaken (using the computer program G power) to determine the number of participants that will be recruited in year two–the beginning of the true research study. To obtain sufficient power (.80) assuming medium effects (f = .15), a sample size of 80 participants are required. The statistical power associated with the sample in the pilot project (n = 15) is .365 (F(3,42) = 1.569).

### Design

A pre/post treatment designed was used in order to monitor the participants' progress and allow for a baseline comparison and also to maximize the applicability of the trial's results to usual care settings. This design also provides preliminary evidence for the group-based intervention which will aid in the design of future randomized control trials (RCT).

### C.H.A.M.P. Research Components

Children and family members completed a number of research-related assessments (see Table S1 in the additional file 1 for a timeline of assessments). Measurements were taken at one or more of the following time points: baseline (i.e., approximately one week before the start of the formal intervention), the first day of the intervention (i.e., beginning of Week 1), two weeks later (i.e., end of Week 2), the last day of the intervention (i.e., end of Week 4), post-intervention (i.e., within one week after completion of the formal program), 3 months post-intervention, 6 months post-intervention, and/or 12 months post-intervention. Additional information pertaining to these measurements is provided in the *Measurement of Main Outcomes *section below.

### Theoretical Model: General

For the past 25 years, family-based behavioral interventions have been shown to produce both short and long term positive results [31]. The family-based behavioral intervention model developed by Epstein and colleagues [31,32] was used as a framework for C.H.A.M.P. That is, it was believed that both family and peer (group) support would contribute to the success of the participants and the overall effectiveness of the program. Additionally, from a group dynamics perspective, a sense of *groupness *develops naturally in any collective in which individuals spend time and interact and communicate with one another. However, group dynamics theory, research, and practice show that process of becoming a more unified group can be improved through the introduction of a number of strategies. As mentioned above, many group dynamics-based strategies have been shown to be highly effective in intervention programs targeting older and/or special populations [19,33,34].

Utilizing these two approaches in combination, several evidence-based group dynamics strategies were implemented at two distinct yet related levels, with two cohorts: (1) children, and (2) family members (i.e., parents or guardians). The specific strategies used at both levels of C.H.A.M.P. are outlined below.

### Theoretical Model: Children's Component

In order to facilitate perceptions of group cohesion and social support, the children were organized into three levels of groups during the 4-week intervention: the whole CHAMP population (*n *= 15), two smaller teams (*n *= 7 and *n *= 8 in each), and six smaller counselor groups (*n *= 2 or 3 in each). The children completed activities in all three levels of groups both during and following the formal intervention.

#### Increasing a sense of distinctiveness

Groups that perceive themselves to be unique or distinctive from other groups develop a stronger sense of cohesiveness. In order to maximize the participants' sense of distinctiveness, group dynamics strategies were used at all three levels of groups. For the whole C.H.A.M.P. group, all children were provided with two purple C.H.A.M.P. t-shirts, a knapsack, and a binder containing handouts, resources, and worksheets (all of which were labelled with a clear and identifiable C.H.A.M.P. logo). Additional strategies used to enhance distinctiveness included emphasizing that these children were the first cohort of participants to take part in this unique and innovative program, and to promote unique C.H.A.M.P. "traditions" including the Olympic Gold Medal Ceremony and the C.H.A.M.P. Talent Show.

As indicated above, the second level of groups were the two C.H.A.M.P. teams. On the first morning of camp, children were divided into the two teams and given time to work as a group to create a team name, logo, cheer, and flag. The team names chosen by the participants were "Purple Rain" and "Golden Sharks".

Finally, as mentioned above, the third level consisted of the C.H.A.M.P. counselor groups. By their very nature, these groups were distinct in that each consisted of a different counselor and two to three campers of roughly the same age and, if possible, sex.

#### Proximity

Research has shown that when group members are brought into close physical proximity with one another, cohesiveness increases; this is the result of increased opportunities for task and social interactions [35]. The C.H.A.M.P. group as a whole had a designated meeting room and group of lockers at the facility in which the program took place. Each child selected a seat in the classroom and a locker where they were able to store their belongings; these remained constant throughout the program.

With regard to the smaller C.H.A.M.P. teams (i.e., "Purple Rain" and "Golden Sharks"), children spent a considerable time together in close proximity during team-based activities, and in many instances, gravitated towards the same roles/and positions during sports and other activities.

Finally, because children were instructed to remain near their counselor at all times, being in close proximity with the counselor and other campers in the counselor groups occurred naturally. For example, children traveled to and from various activities and locations (e.g., walking to and from the park, swimming pool, locker room, fields, etc.) in their small counselor groups. They also ate lunch together at the same table every day, met for "team time" at the same table every morning and afternoon, and sat in their counselor groups for educational sessions.

#### Ongoing group interactions and group-based activities

As mentioned previously, C.H.A.M.P. activities were conducted at all three levels of groups. Several activities were carried out with the entire C.H.A.M.P. group (and thus, were not team-based); these included the C.H.A.M.P. Talent Show which took place on the last day of camp, educational sessions, various aerobic (e.g., dance), water (e.g., synchronized swimming), and/or games-based (e.g., obstacle course) activities, and the supervised physical activities that took place during weekend family sessions and follow-up booster sessions. Additionally, children worked together as a large group during the C.H.A.M.P. Olympics (in which an activity-based relay took place) and some field trips (e.g., gymnastics).

Many of the activities that took place during the 4-week program related to the second level of groups in that they were team-based. The two teams competed in friendly competitions in a wide range of team-based sports and activities (e.g., soccer, floor hockey, basketball, water polo, etc.). For many of these activities, participants carried their team flag, encouraged their teammates, and chanted their team name and song.

A number of activities were carried out with the smaller counselor groups. From a logistical/safety perspective, counselor groups remained together for all field trips (e.g., bowling, mini-putt, water-park, batting cages, rock climbing, etc.). Additionally, counselor groups worked together during sessions in which techniques, skills, and drills were reviewed and practiced with each child. Throughout the day, the counselor groups engaged in a number of other activities together (some of which have been described) including: eating lunch together, working on various crafts and exercises, travelling to and from various locations, engaging in homework and problem-solving discussions, and working on C.H.A.M.P. passports and "3 Things" worksheets (discussed below).

#### Goal setting

Establishing and working toward the attainment of collective and individual goals serves to enhance group cohesiveness. All C.H.A.M.P. participants and counselors attended a goal setting workshop (led by the Principal Investigator) during which the importance of setting S.M.A.R.T. (specific, measurable, attainable, realistic, and time-based) goals was emphasized. Additionally, the usefulness of setting individual and group goals–both at home and at camp–was discussed.

At the beginning of each week, the children worked within their two smaller teams to establish weekly goals for their group. These goals related to physical activity (i.e., go for a 30 minute walk at home each night) and nutrition (i.e., bring one vegetable and one fruit to camp each day) either in the home or camp environment. Children completed home-based physical activity and nutrition logs, and parents/guardians were asked to initial the logs each night. Counselors kept track of each child's progress, and tallied the behaviors for both groups on a daily basis. During lunch each day, a specific number of "kilometres" was awarded to each team for their combined behaviors (for example, 15 minutes of walking was equal to one kilometre). Using the "C.H.A.M.P. Road Trip Across Ontario" map that was strategically placed at the front of the classroom, each team was moved along the map according to the total number of kilometres they had earned as a group (each group was represented by a small toy car). At the end of the week, the group that made the most progress (i.e., covered the most distance across the province) was rewarded with a small prize.

Finally, all children were provided with weekly C.H.A.M.P. "passports" that corresponded to the theme of the week (i.e., sports, nutrition, Olympics, adventure). These passports included a section pertaining to "weekend goals", with spaces for three individual goals. Children were encouraged to work with their counselor and teammates in the smaller counselor groups to set appropriate (and S.M.A.R.T.) goals, and to be accountable for meeting these objectives. The individuals in the counselor groups also worked together to create colourful signs for each child's weekend goals that could be placed in a visible location in the home environment (e.g., on a fridge or bulletin board).

#### Ongoing communication, feedback, and social support

An important component of C.H.A.M.P. was the implementation of frequent group-based discussions pertaining to health behaviors, self-esteem, and specific challenges and successes that children experienced throughout the program; these discussions took place regularly at all three levels of groups. For example, structured group-based discussions were held for 5 to 10 minutes for all C.H.A.M.P. participants following each activity/educational session that took place. These sessions were led by counselors, and were used as de-briefing sessions in which children were asked: (1) to share their thoughts and ask questions about the activity/session; (2) what was positive about the activity/session; (3) what was difficult about the activity/session; and (4) how this activity/information could be applied/continued in the home environment. Counselors also offered constructive feedback during this time. Additionally, as mentioned above, children worked towards the completion of one C.H.A.M.P. passport per week. One section of the passport, entitled "My Friends", required children to learn about and communicate with other campers. For example, children were asked to "find out from three fellow C.H.A.M.P. members what their favourite things to do are" or to "recognize a fellow C.H.A.M.P. member for their good performance or attitude". Once a child completed a specific task, his or her counselor reviewed the information and stamped the appropriate section of the child's passport.

Also pertaining to the first level of groups (i.e., the C.H.A.M.P. group as a whole), a number of life coaching activities were introduced throughout the program, all of which focused on self-esteem, group interaction, and social support. For example, in one activity entitled "The Fabulous Me", each child wrote down one positive characteristic about every other child on a sticky note. Once this was complete, children took turns standing in front of their peers while the other children read their positive comments and "stuck" them on the individual at the front of the room (possibly not surprsingly, most children chose to "wear" their positive comments all day). A "buddy system" was also introduced whereby children were assigned to a partner and asked to sit with him or her on the bus, both on the way to and home from camp. This was implemented in order to further increase interaction among the children.

At the team level, cheering for and encouraging teammates (i.e., saying "you can do it" or using high fives) became an accepted–and required–part of the C.H.A.M.P. culture. Counselors encouraged this praise, and offered their own positive feedback on a regular basis. During the last weekend session, team-based focus groups (i.e., one 60 minute focus group for each team) were conducted by members of the research team to facilitate communication and to further explore the perceptions and experiences of the children involved in the program.

Finally, for the smaller counselor groups, children and counselors participated in 15 minutes of "team time" every morning and afternoon, where they communicated about the successes, challenges, and other positive or negative thoughts the children experienced throughout the program. Additionally, every afternoon, all children completed a "3 Things I Did Well Today" worksheet in their counselor groups. These worksheets required children to think about the day, and to discuss their positive experiences with their counselor and the other children in their small groups. They then wrote down three different (and specific) things they did well, and ended the day on a positive note.

#### Decision making

Research in sport has shown that when teammates work together to arrive at a collective decision (that is, using a democratic style of decision making), perceptions of both task and social cohesion increase [36]. At various times throughout the program, children were given the opportunity to work as a group to make their own decisions with regard to a number of camp-related issues and activities. For example, for the C.H.A.M.P. group as a whole, the schedule allowed for several "camper's choice" time slots where the participants discussed (as a group and with counselors) the activity in which they would like to engage. This allowed the children to feel as if they had control over the activities they participated in, and provided them with an opportunity to work together to reach a consensus. In both the smaller teams and counselor groups, children were regularly encouraged to work together to discuss and make choices pertaining to issues and activities related to goal setting, roles for the talent show, positions for games-based activities, and so on.

#### Sacrifice behavior

When individuals make sacrifices for their groups, they psychologically invest in that group and develop stronger perceptions of its cohesiveness. Throughout the 4-week intervention, children were required to make sacrifices for other members of all three groups to which they belonged. For example, due to the wide age range of participants (i.e., ages 8 to 14) some of the older children did not want to participate in the activities that the younger children enjoyed, and vice versa. These situations necessitated group discussions, compromise, and individual sacrifices. Another (voluntary) use of sacrifice behavior by the children was the fact that several older children willingly and regularly sacrificed their own time to assist the younger children in a number of situations (e.g., escorting them to the washroom and water fountain, etc.) and with certain activities (e.g., skills, swimming techniques, etc.).

### Theoretical Model: Family (parent or guardian) Component

As mentioned previously, all weekend family education sessions were conducted in a group setting. A number of group-based protocols were incorporated into this aspect of C.H.A.M.P.

#### Goal setting

During the second weekend session, families participated in a group-based goal setting workshop led by the Principal Investigator. Similar to the goal setting session introduced to the children, a presentation was conducted in which the importance of setting S.M.A.R.T. goals was emphasized, as were the issues of short and long term goals, accountability, family involvement, and family commitment. Families (parents/guardians and their children]) worked together to complete a "C.H.A.M.P. Family Action Plan" that included the following prompts: (a) These are the reasons my family is involved in C.H.A.M.P.; (b) Our family's top 3 goals for the next 4 weeks are...; and (c) State the actions your family will take over the next 4 weeks to reach your goals. Insofar as the last element is concerned (i.e., point [c] above), families were asked to outline: the *changes *they intended to make in their physical activity and/or nutrition behaviors, the *strategies *they intended to use to accomplish these goals, and the *resources *they intended to use to accomplish these goals. The last section required a parent or guardian to complete the following statement: "*On _________(date) I will be prepared to report back on my family's progress to _______ (name of C.H.A.M.P. "buddy"). At that time we will talk about our successes and challenges and set new goals to continue on our paths of healthy living. (Phone #: ______)*." Finally, all family members signed the document and a copy of the worksheet/contract was given to the researchers to further enhance accountability.

#### Information sharing

During the family education sessions and the follow-up booster sessions, weekly messages, resources, and handouts regarding healthy living, ideas for physical activity, and motivation were provided to families (parents were provided with C.H.A.M.P. binders in which to store this information). These sessions also allowed parents and guardians to openly share and discuss their successes and difficulties in the home environment, and to ask questions to other group members or health care professionals. To further enhance communication among children, family members, and camp/research staff during the 4-week intervention, a weekly newsletter containing pictures, information about the previous week, health-related information, and upcoming events was provided to families. Each newsletter also contained a page entitled "The Fabulous You", which was dedicated to recognizing an outstanding accomplishment for each child during the previous week. As mentioned previously, families also received bi-monthly newsletters containing health- and program-related information following completion of the formal 4-week intervention.

#### Ongoing communication, feedback, and social support

One of the primary purposes of both the family-based education sessions and the follow-up booster sessions was to provide families with an opportunity to meet, communicate, and interact with other individuals in a similar situation to themselves. Thus, all aspects of the family-based sessions centred around communication, feedback (where necessary), and social support. For example, parents were asked to share their contact information with one other C.H.A.M.P. family to enhance accountability for their family goals, and to increase communication, interaction, and support among families. Parents also participated in focus groups during which they were encouraged to openly share their perceptions of and experiences with the program. Finally, parents provided weekly feedback (both verbally and in written form via self-report questionnaires) to researchers about their involvement in the program.

Open lines of communication were also maintained among counselors, program staff, and families throughout and following the 4-week program. When necessary, parents were provided with verbal or written updates regarding their child, and families were also given contact information for two counselors and several members of the research team. Following program involvement, families were contacted by program staff on a regular basis as a means of communication and support, and to relay friendly reminders for program-related events, sessions, and research assessments.

#### Collective problem solving

For all family-based sessions, barriers to physical activity and healthy eating were identified and group discussions were implemented to create solutions for overcoming such challenges.

#### Family-based physical activity

To increase physical activity, group support, and family commitment, all families were provided with a one-month membership (for all family members) at the YMCA in London, Ontario. In addition, one of the follow-up booster sessions consisted of a family-based aerobics/strength training session for children, siblings, and guardians. Finally, families were provided with coupons and information for family-friendly activities (e.g., family skating time, hiking trails, bike paths, etc.) that could be utilized following involvement in the 4-week program.

### Measurement of Main Outcomes

A mixed methods (i.e., quantitative and qualitative) approach was taken in the collection and evaluation of data generated from the intervention and follow-up assessments. Several outcomes were assessed at various time points including standardized body mass index (BMI-*z*), waist circumference, lean body mass, body fat percentage (all of which were measured via dual x-ray absorptiometry [DXA] scans), and specific fitness indices (i.e., exercise heart rates, resting heart rate, and distance run/walked during the Cooper 12-minute walk/run test [37]. Objective levels of physical activity were also assessed using Actical^® ^Accelerometers (MiniMitter, Oregon). Fasting blood sample measurements yielded values for total cholesterol, high density lipoprotein cholesterol (HDL-c), low density lipoprotein cholesterol (LDL-c), triglycerides, insulin, serum glucose, and HOMA-IR. Additionally, vessel wall imaging (VWI) was conducted on all children to monitor plaque formation, vessel elasticity, and capillary function, thus providing an estimate of cardiovascular health. As mentioned previously, semi-structured focus groups were also conducted with children and parents to gain qualitative information pertaining to participants' experiences with and the perceived impact of the program. Finally, self-report measures included children's health-related quality of life (measured via the PedsQL 4.0) [38-40], the Physical Activity Questionnaire for Children (PAQ-C) [41], questions pertaining to the Theory of Planned Behavior constructs [42], task and barrier self-efficacy for physical activity (using an adapted version of the Self-Efficacy Scale [43], and perceptions of belongingness (i.e., cohesion). For the purpose of the present paper, only the measure pertaining to cohesion are discussed in detail.

#### Cohesion measure

Cohesion was measured using an 18 item questionnaire developed specifically for the present research and modified from the Sports Cohesiveness Questionnaire [44]. The first two questions had children identify their counselor and C.H.A.M.P. team (i.e., Golden Sharks or Purple Rain). The third question asked children to "*Rate each of your C.H.A.M.P. camp-mates on how much of a friend they are to you*". Children were shown pictures of all C.H.A.M.P. participants, and asked to rate each child on a scale from 1 ("very good friend") to 5 ("not at all my friend"). The remaining 15 questions were answered on a five-point response scale (1 = highest, 5 = lowest), with a corresponding graphic scale containing faces with a range of emotional expressions (1 = smiling face, 5 = frowning face) adapted from Wong and Baker [45]. These questions were designed to assess children's perceptions of friendship (3 items), importance of the group (3 items), fitting in (3 items), enjoyment (3 items), and closeness with other group members (3 items). More specifically, items pertained to the feelings of unity or closeness of the participants towards *the total C.H.A.M.P. population *(e.g., "Compared to other groups or teams that you belong to, how important is being part of the C.H.A.M.P. team [as a whole] to you?"), *the C.H.A.M.P. teams *(e.g., "How much do you feel like you fit in with your specific CHAMP team [Purple Rain or Golden Sharks]?), and *the C.H.A.M.P. counselor groups *(e.g., "How good do you think the teamwork is in your C.H.A.M.P. counselor group?"). As can be seen in Table S1 (additional file 1), the cohesion measure was administered at Week 2 of the intervention, the last day of C.H.A.M.P. (Week 4), and at 3-, 6-, and 12-month follow-up assessments. Data were collected at these time periods to determine the effectiveness of the group dynamics strategies used during the intervention, and whether cohesion levels were maintained once the formal intervention finished.

## Discussion

In summary, this article has provided a rationale for the creation of C.H.A.M.P., a background in the area of childhood obesity, and an overview of the group dynamics strategies used to facilitate cohesion among children and family members. All of the information needed to create and implement a similar group-based program has been discussed: study design, components of the intervention, recruitment, and measurement procedures. Although data analyses from Year 1 are not complete, preliminary results appear promising and anecdotal reports from children, guardians, and counselors have been positive. Additionally, the first year of this pilot project has provided valuable information regarding what aspects of the intervention appear to be most and least effective. The results of the analyses will help further the knowledge in family- and group-based interventions aimed at the treatment of overweight and obese children. Results of the intervention will be available in late 2009, and will be disseminated to relevant community and government organizations, and published in academic journals. In the meantime, it is hoped that the issues discussed provide guidance to those undertaking similar trials with children. Ultimately, it is our goal to utilize the results of the 2-year pilot project to offer C.H.A.M.P. on an annual basis with a larger number of children and families across a broader range of ages, settings, and communities.

## Competing interests

The authors declare that they have no competing interests.

## Authors' contributions

SB, JI, RP, HP, and KS were all involved in the development of the program. They provided support and expertise related to the design and implementation of the study. LM, SB, AC, and SS participated in the writing of the manuscript, while JI, RP, HP, and KS provided revisions and comments, and approved the final version. All authors read and approved the final version of the manuscript.

## Pre-publication history

The pre-publication history for this paper can be accessed here:



## Supplementary Material

Additional file 1 **Table S1.** Click here for file

## References

[B1] Statistics Canada (2006). Overweight and obesity among children and youth. Health Reports.

[B2] Deakin T, McShane CE, Cade JE, Williams RD (2005). Group based training for self-management strategies in people with type 2 diabetes mellitus. Cochrane Database of Systematic Reviews.

[B3] Summerbell CD, Ashton V, Campbell KJ, Edmunds L, Kelly S, Waters E (2003). Interventions for treating obesity in children. Cochrane Database of Systematic Reviews.

[B4] Carey VJ, Walters EE, Colditz GA, Soloman CG, Willet WC, Rosner BA, Speizer FE, Manson JE (1997). Body fat distribution and risk of non-insulin-dependent diabetes mellitus in women. The Nurses' Health Study. American Journal of Epidemiology.

[B5] Chan JM, Rimm EB, Colditz GA, Stampler MJ, Willet WC (1994). Odesity, fat distribution, and weight gain as risk factors for clinical diabetes in men. Diabetes Care.

[B6] Davis S, Gomez Y, Lambert L, Skipper B, Williams CK, Kimm SYS (1993). Primary prevention of obesity in American Indian children. Prevention and Treatment of Childhood Obesity.

[B7] Eisenmann JC, Gentile DA, Welk GJ, Callahan R, Strickland S, Walsh M, Walsh DA (2008). SWITCH: rationale, design, and implementation of a community, school, and family-based intervention to modify behaviors related to childhood obesity. BMC Public Health.

[B8] Fiztgibbon ML, Stolley MR, Dyer AR, VanHorn L, KauferChristoffel K (2002). A community-based obesity prevention program for minority children: rationale and study design for Hip-Hop to Health. Journal of preventative medicine.

[B9] Singh AS, Chin A, Paw MJM, Kremers SPJ, Visscher TLS, Brug J, van Mechelen W (2006). Design of the Dutch Obesity Intervention in Teenagers (NRG-DOiT): systematic development, implementation and evaluation of a school-based intervention aimed at the prevention of excessive weight gain in adolescents. BMC Public Health.

[B10] Wilfley DE, Tibbs TL, Van Buren DJ, Reach KP, Walker MS, Epstein LH (2007). Lifestyle interventions in the treatment of childhood overweight: a meta-analytic review of randomized controlled trials. Health Psychology.

[B11] American Dietetic Association. Position of the American Dietetic Association (2006). individual-, family-, school-, and community-based interventions for pediatric overweight. Journal of the American Dietetic Association.

[B12] Ritchie LD, Welk G, Styne D, Gerstein D, Crawford PB (2005). Family environment and pediatric overweight: what is a parent to do?. J Am Diet Assoc.

[B13] Epstein LH, Wing RR (1987). Behavioral treatment of childhood obesity. Psychology Bulletin.

[B14] Paffenbarger RS, Hyde RT, Dishman RK (1988). Exercise adherence, coronary heart disease, and longevity. Exercise adherence: Its impact on public health.

[B15] Bouchard C, Shepard RJ, Stephens T, Sutton JR, McPerson DB (1990). Exercise, Fitness and Health.

[B16] Camacho TC, Roberts RE, Lazarus NB, Kaplan GA, Cohen RD (1990). Physical activity and depression: Evidence from the Alameda County Study. American Journal of Epidemiology.

[B17] Plante TG, Seraganian P (1993). Aerobic exercise in the prevention and treatment of psychopathology. Exercise psychology: The influence of physical exercise on psychological processes.

[B18] Blair SN, Kohl HW, Paffenbarger RS, Clark DG, Cooper KH (1989). Physical fitness and all-cause mortality: A prospective study of healthy mean and women. Journal of the American Medical Association.

[B19] Brawley LR, Rejeski WJ, Lutes L (2000). A Group-Mediated Cognitive-Behavioral intervention for Increasing Adherence to Physical Activity in Older Adults. Jounral of Applied Biobehavioral Research.

[B20] Estabrooks PE, Carron AV (1999). Group cohesion in older adult exercisers: Prediction and intervention effects. Journal of Behavioral Medicine.

[B21] Rejeski WJ, Brawley LR, Ambrosius WT, Brubaker PH, Focht BC, Foy CG, Fox LD (2003). Older adults with chronic disease: Benefits of group-mediated counseling in the promotion of physically active lifestyles. Health Psychology.

[B22] Watson JD, Martin Ginis KA, Spink KS (2004). Team building in an exercise class for the elderly. Activities, Adaptation & Aging.

[B23] Cramp AG, Brawley LR (2006). Moms in montion: a group-mediated cognitive-behavioral physical activity intervention. International Journal of Behavioral Nutrition and Physical Activity.

[B24] Spink KS, Carron AV (1993). The effects of team building on the adherence patterns of female exercise participants. Journal of Sport and Exercise Psychology.

[B25] Baumeister RF, Leary MR (1995). The need to belong: Desire for interpersonal attachments as a fundamental human motivation. Psychological Bulletin.

[B26] Eaton SB, Konner M (1985). Paleolithic nutrition: A consideration of its nature and current implications. International Bibliographic Information on Dietary Supplements.

[B27] Van Vugt M, Schaller M (2008). Evolutionary approaches to group dynamics: An introduction. Group Dynamics: Theory, Research, and Practice.

[B28] Carron AV, Burke SM (2005). Context and physical activity. The influence of others. Sport and Exercise Psychology Review.

[B29] Epstein LH (1996). Family-based behavioral intervention for obese children. Int J Obes Rela Metab Disord.

[B30] Sallis JF, Simons-Morton BG, Stone EJ, Corbin CB, Epstein LH, Faucette N, Iannotti RJ, Killen JD, Klesges RC, Petray CK (1992). Determinants of physical activity and interventions in youth. Med Sci Sports Exerc.

[B31] Epstein LH, Paluch RA, Roemmich JN, Beecher MD (2007). Family-based obesity treatment, then and now: twenty-five years of pediatric obesity treatment. Health Psychol.

[B32] Epstein LH (1996). Family-based behavioral intervention for obese children. Int J Obes Relat Metab Disord.

[B33] Cramp AG, Brawley LR (2006). Moms in motion: a group-mediated cognitive-behavioral physical activity intervention. International Journal of Behavioral Nutrition and Physical Activity.

[B34] Rejeski WJ, Foy CG, Brawley LR, Brubaker PH, Focht BC, Norris JL, Smith ML (2002). Older adults in cardiac rehabilitation: a new strategy for enhancing physical function. Medicine and Science in Sports and Exercise.

[B35] Carron AV, Hausenblas HA, Eys MA (2005). Group dynamics in sport.

[B36] Brawley LR, Carron AV, Widmeyer WN (1993). The influence of the group and its cohesiveness on perceptions of group goal-related variables. Journal of Sport & Exercise Psychology.

[B37] Cooper KH (1968). A means of assessing maximal oxygen intake: Correlation between field and fitness testing. Journal of the American Medical Association.

[B38] Schwimmer JB, Burwinkle TM, Varni JW (2003). Health-related quality of life of severely obese children and adolescents. Journal of the American Medical Association.

[B39] Varni JW, Seid M, Kurtin PS (2001). The PedsQL 4.0: Reliability and validity of the Pediatric Quality of Life Inventory Version 4.0 Generic Core Scales in health and patient populations. Medical Care.

[B40] Varni JW, Seid M, Rode CA (1999). The PedsQL: Measurement model for the Pediatric Quality of Life Inventory. Medical Care.

[B41] Crocker PR (1997). Measuring general levels of physical activity: preliminary evidence for the Physical Activity Questionnaire for Older Children. Medicine & Science in Sports & Exercise.

[B42] Ajzen I (2002). Perceived behavioral control, self-efficacy, locus of control, and the theory of planned behavior. Journal of Applied Social Psychology.

[B43] McAuley E, Mihalko SL, Duda JL (1998). Measuring exercise-related self-efficacy. Advances in sport and exercise psychology measurement.

[B44] Martens R, Landers D, Loy J (1972). Sports cohesiveness questionnaire.

[B45] Wong D, Baker C (1988). Pain in children: comparison of assessment scales. Pediatric Nursing.

