# Supplementary material for: The use of group dynamics strategies to enhance cohesion in a lifestyle intervention program for obese children
Source: BMC Public Health. 2009 Jul 31;9:277. doi: 10.1186/1471-2458-9-277 (PMC2723112; doi:10.1186/1471-2458-9-277)
Supplement: Additional file 1 — Table S1. [file 1471-2458-9-277-S1.doc]

Table 1

Measurement timeline for C.H.A.M.P.

| Type of Measurement | Baseline | First day of CHAMP | 2 weeks into CHAMP | Last day of CHAMP | Post Intervention Assessment | 3 Month Follow-up | 6 Month Follow-up | 12 Month Follow-up |
| --- | --- | --- | --- | --- | --- | --- | --- | --- |
| Body composition (DXA) | X |  |  |  | X |  | X | X |
| Health-related quality of life^1^ | X |  |  |  | X | X | X | X |
| Blood profiles/ medical assessment | X |  |  |  | X |  | X | X |
| Waist circumference | X |  |  |  | X |  | X | X |
| Actical^2^ | X |  |  |  | X | X | X | X |
| Vessel Wall Imaging | X |  |  |  | X |  | X | X |
| Fitness indices^3^ |  | X |  | X |  |  | X | X |
| Subjective physical activity questionnaire ^4^ |  | X |  | X |  | X | X | X |
| Theory of Planned Behavior^5^ |  | X |  | X |  | X | X | X |
| Self-Efficacy^6^ |  | X |  | X |  | X | X | X |
| Cohesion^7^ |  |  | X | X |  | X | X | X |
| Focus Groups |  |  |  |  | X |  |  | X |

*Notes*

^1^ Peds QL 4.0 (Varni, 1999)

^2^ Actical® Accelerometers (MiniMitter, Oregon)

^3^ The Cooper 12 minute walk/run fitness test (Cooper, 1968)

^4^ Physical Activity Questionnaire for Children (PAQ-C; Crocker et al., 1997)

^5^ Theory of Planned Behavior Constructs (Azjen, 2002)

^6^ Adapted version of the Self Efficacy Scale (McAuley & Mihalko, 1998)

^7^ Cohesion items (Marten, Landers & Loy, 1972), graphic scale adapted from (Wong & Baker, 1988)
